# Pygmy Populations Seronegative for Marburg Virus

**DOI:** 10.3201/eid1101.040377

**Published:** 2005-01

**Authors:** Matthias Borchert, Sabue Mulangu, Robert Swanepoel, Antoine Tshomba, Afongenda Afounde, Amayo Kulidri, Jean-Jacques Muyembe-Tamfum, Patrick Van der Stuyft

**Affiliations:** * Institute of Tropical Medicine, Antwerp, Belgium;; † Institut de Recherche Biomédicale, Kinshasa, Democratic Republic of Congo;; ‡ National Institute for Communicable Diseases, Johannesburg, South Africa;; § Hôpital Général de Kilo-Moto, Watsa, Democratic Republic of Congo;; ¶ Ministry of Health, Democratic Republic of Congo

**Keywords:** letter, Marburg virus disease, seroprevalence, epidemiology

**To the Editor:** A serosurvey was conducted in Durba, a mining village near Watsa, northeastern Democratic Republic of Congo, the epicenter of Marburg hemorrhagic fever (MHF) outbreaks in 1994 and 1998–2000 (*1**–**3*). In this survey, Bausch et al. found a prevalence of anti-Marburg immunoglobulin (Ig) G of 0.35% (2 of 565) in the nonmining population, but a prevalence of 3.75% (13 of 347) in miners. Mine work was an independent risk factor for seropositivity for anti-Marburg IgG (*1*). Given that widespread secondary transmission could not be documented in the seropositive miners, primary transmission from the unknown reservoir likely occurred in the mines where rodent, shrew, bat, and other fauna were abundant. No evidence of Marburg virus (MBGV) infection was found in samples from small mammals, amphibians, and arthropods collected in and around Gorumbwa mine (R. Swanepoel, pers. comm.); the origin of the MHF outbreak remained unknown.

We hypothesized that the MBGV reservoir's habitat might not be limited to gold mines around Durba, but may exist in caves or forests in the wider Watsa area. As hunter-gatherers, pygmies enter caves for shelter and are in frequent contact with wild animals and body fluids of butchered game. Earlier studies found that pygmies were seropositive for filoviruses significantly more often than subsistence farmers (for filoviruses [4,5], for Ebola but not Marburg [6]). We conducted a seroprevalence study to verify whether pygmies living in the Watsa area constitute another population at risk for primary transmission of MBGV.

The Watsa area's population (≈180,000) includes 4,000 pygmies living predominantly in its southern parts (*1*). The pygmies live seminomadically in the forest, occasionally leaving to exchange goods with the sedentary Bantu population.

We invited the pygmy population to meet with our study representatives at sites 50–90 km from Durba. Three hundred persons volunteered during a 5-day period. After informed verbal consent was obtained, the study participants were interviewed, and a blood sample was taken from each volunteer. For operational reasons, we excluded children <10 years old. According to local customs, men received small quantities of salt and soap and women received an item of second-hand clothing as an appreciation for their efforts. Ethical clearance was obtained by the ethics committee of the Institute of Tropical Medicine in Antwerp and the representative of the Ministry of Health in Watsa.

The study questionnaire was similar to one used in the Durba 1999 survey; we did not maintain a recall period of 1 year for exposures related to medical treatment, as this did not appear to be a meaningful time span for the pygmies. Procedures for collecting and handling blood samples were similar to the Durba survey, and the same laboratory tests were applied. Serum samples were considered positive only if they were positive for Marburg IgG in both enzyme-linked immunosorbent assay and indirect immunofluorescence assay (IFA) (*1*).

The study participants originated from 39 different settlements. Their median age was 30 years (range 10–75; q1 20, q3 40); half of them were males. Most study participants reported activities (hunting 60%, entering caves 98%) and contacts with wild animals (rodents 79%, bats 78%, monkeys or apes 99%) thought to be risk factors for the primary transmission of filoviruses. Whenever noticeable differences existed between the sexes, men tended to be exposed more frequently than women, often significantly so. Pygmies were significantly more exposed to wild animals than the nonmining general population; the difference was particularly large concerning contact with bats (Table). From one fourth to one third of study participants reported a direct or potential contact with someone with a febrile hemorrhagic syndrome. Women were more frequently exposed to these risk factors for secondary transmission in the household or community than men, sometimes significantly so; pygmies were less exposed to these risk factors than the nonmining general population (Table). Almost all study participants had been exposed at least once in their life to invasive modern or traditional medical treatment, including injections and scarification, by which an iatrogenic secondary transmission could have occurred.

**Table:** Frequency of risk factors for Marburg hemorrhagic fever in pygmies and nonmining general population residing in the Watsa Health Zone, Democratic Republic of Congo

Risk factors	Male pygmies (n = 150) (%)	Female pygmies (n = 150) (%)	p*	Pygmy population (n = 300) (%)	Nonmining population (n = 553 to 569)† (%)	p*
Primary transmission risk factors						
Subsistence activities						
Hunting	100	20	< 0.001	60	–	–
Entering caves	98	99	0.7	98	–	–
Contacts with wild animals						
Rodents						
Touched	85	59	< 0.001	72	53	< 0.001
Eaten‡	42	43	0.9	42	34	0.02
Bitten by	33	27	0.3	30	26	0.15
Any contact	88	71	< 0.001	79	65	< 0.001
Bats						
Touched	81	68	0.008	75	16	< 0.001
Eaten‡	59	47	0.04	53	3	< 0.001
Bitten by	23	15	0.06	19	0.9	< 0.001
Any contact	83	72	0.02	78	18	< 0.001
Monkeys, apes						
Touched	99	83	< 0.001	91	59	< 0.001
Eaten‡	97	96	0.8	96	79	< 0.001
Bitten by	6	5	0.6	5	8	0.2
Any contact	99	97	0.1	98	84	< 0.001
Any wild animals	99	98	0.3	99	90	< 0.001
Secondary transmission risk factors						
Contact with FHS§ patient						
In the same household with FHS patient	19	25	0.3	22	25	0.4
In the same room with FHS patient	11	20	0.04	16	22	0.03
Worked with FHS patient	16	25	0.06	20	28	0.02
Participated in funeral of FHS patient	19	25	0.2	22	44	< 0.001
Touched FHS patient	15	23	0.06	19	32	< 0.001
Touched blood, urine, feces of FHS patient	10	13	0.5	11	7	0.03
Touched remains of FHS patient	11	19	0.05	15	10	0.02
Any contact	27	36	0.1	32	58	< 0.001
Any direct contact (touched)	22	31	0.09	26	34	0.02
Invasive medical treatment¶						
Ever received injection	85	90	0.2	88	–	–
Ever received surgical or obstetric care	52	31	< 0.001	41	–	–
Any invasive medical treatment ever	93	93	–	93	–	–
Traditional treatment						
Ever had scarification	99	97	0.4	98	–	–

*Using chi-square test. †Variation in sample size due to missing data. ‡Bush meat often is smoked, grilled, or cooked; exposure to viable virus may therefore be more likely to happen during preparation of such meat for consumption than during consumption itself. §FHS (febrile hemorrhagic syndrome): severe illness with high fever and bleeding from the nose, mouth or anus. ¶Includes circumcision, abscess incision, and other minor intervention.

Thirty-seven percent of the study participants reported having experienced a febrile hemorrhagic syndrome at least once in their life, men more often than women (n = 236; 45% versus 28%, chi-square test: p = 0.006). All serum samples, however, were negative for anti-Marburg IgG; the prevalence of anti-Marburg IgG in the pygmy population (0.0%; exact binomial one-sided 97.5% confidence intervals [CI] 0.00%–1.2%) was similar to that in Durba's nonmining population (0.35%; 95% CI 0.04%–1.3%), significantly lower than in Durba's mining population (3.7%; 95% CI 2.0%–6.3%), and as low as, or even lower than, that in other populations in sub-Saharan Africa, where a seroprevalence was found in 0% to 1.7% in 15 studies. Only 2 studies from the Central African Republic and Uganda found a higher seroprevalence (3.2% and 4.5%, respectively; all studies are referenced [1]). In studies conducted before the 1999 Durba survey, the presence of anti-Marburg IgG has been determined by only the less specific IFA; this may explain why we have found a lower prevalence in our study population than reported from certain other locations in sub-Saharan Africa.

We reject our study hypothesis that pygmies residing in the Watsa area are a second population at risk for MHF compared with the nonmining sedentary population. We conclude that the absence of anti-Marburg IgG in the pygmy population reflects the virtual absence of MBGV circulation in the reservoir, the absence of the reservoir in the pygmies' environment, the absence of exposure to the reservoir, or any combination of these. The MHF outbreaks in Durba and Watsa in 1994 and 1998–2000 apparently did not impact the study population. The frequent occurrence of febrile hemorrhagic syndrome was almost certainly due to a different origin than MBGV and may not have been of viral origin at all.

An alternative explanation for the absence of antibodies would be that the case-fatality proportion was higher than observed during the outbreaks in Durba and Watsa (71%) (*3*). However, there is no reason to assume that pygmies who contract MHF would die more frequently than diseased gold diggers and their family members. Access to basic clinical care is similar in both groups, and this care has a limited effect on the case-fatality proportion.

Another alternative explanation would be that anti-Marburg IgG wanes and becomes undetectable soon after infection. However, all 17 survivors of confirmed MHF in the 1994 and 1998–2000 Durba and Watsa outbreaks with whom we could follow up are still seropositive 22–102 months after onset of disease (M. Borchert, unpub. data).

Our study participants were volunteers who could reach the meeting points along the main road with relative ease. Primary transmission of MBGV may occur more frequently in pygmies living deeper in the forest, but even in those who reached the meeting point and participated in our study, the prevalence of risk factors was very high. Reported exposure patterns correspond to the traditional distribution of tasks such as men hunting and women caring for sick relatives, which lends credibility to our interview data. Gonzalez et al. did not find a significant difference for the risk of filovirus infection between pygmies living in savannah and forest areas (*6*). That the study used volunteers might also have caused seroprevalence to be underestimated if those who rightfully believed they had had MHF in the past, chose not to take part in the study. However, the proportion of study participants reporting to ever have had a febrile hemorrhagic syndrome was high, and MHF was not stigmatized in the study setting. We therefore believe a selection bias is unlikely.

Despite the MHF epidemics in Durba and Watsa in 1994 and 1998–2000, the prevalence of anti-Marburg IgG in the pygmy population of Watsa was as low as, or lower than, that in Durba's nonmining sedentary population, and that in most other populations in sub-Saharan Africa where serosurveys have been conducted. Infection with MBGV appears to be rare in the pygmy population of the Watsa area. During the 1998–2000 outbreak, primary transmission of MBGV was apparently limited to gold mines around Durba. While the location where primary transmission occurred now appears to be well ascertained, the reservoir species at the origin remains unknown.
